# ProDeGe: a computational protocol for fully automated decontamination of genomes

**DOI:** 10.1038/ismej.2015.100

**Published:** 2015-06-09

**Authors:** Kristin Tennessen, Evan Andersen, Scott Clingenpeel, Christian Rinke, Derek S Lundberg, James Han, Jeff L Dangl, Natalia Ivanova, Tanja Woyke, Nikos Kyrpides, Amrita Pati

**Affiliations:** 1 Prokaryotic Super Program, Department of Energy Joint Genome Institute, Walnut Creek, CA, USA; 2 Department of Biology and Curriculum in Genetics, University of North Carolina, Chapel Hill, NC, USA; 3 Department of Biology and Howard Hughes Medical Institute, Curriculum in Genetics, University of North Carolina, Chapel Hill, NC, USA

## Abstract

Single amplified genomes and genomes assembled from metagenomes have enabled the exploration of uncultured microorganisms at an unprecedented scale. However, both these types of products are plagued by contamination. Since these genomes are now being generated in a high-throughput manner and sequences from them are propagating into public databases to drive novel scientific discoveries, rigorous quality controls and decontamination protocols are urgently needed. Here, we present ProDeGe (Protocol for fully automated Decontamination of Genomes), the first computational protocol for fully automated decontamination of draft genomes. ProDeGe classifies sequences into two classes—clean and contaminant—using a combination of homology and feature-based methodologies. On average, 84% of sequence from the non-target organism is removed from the data set (specificity) and 84% of the sequence from the target organism is retained (sensitivity). The procedure operates successfully at a rate of ~0.30 CPU core hours per megabase of sequence and can be applied to any type of genome sequence.

Recent technological advancements have enabled the large-scale sampling of genomes from uncultured microbial taxa, through the high-throughput sequencing of single amplified genomes (SAGs; Rinke *et al.*, 2013; Swan *et al.*, 2013) and assembly and binning of genomes from metagenomes (GMGs; Cuvelier *et al.*, 2010; Sharon and Banfield, 2013). The importance of these products in assessing community structure and function has been established beyond doubt (Kalisky and Quake, 2011). Multiple Displacement Amplification (MDA) and sequencing of single cells has been immensely successful in capturing rare and novel phyla, generating valuable references for phylogenetic anchoring. However, efforts to conduct MDA and sequencing in a high-throughput manner have been heavily impaired by contamination from DNA introduced by the environmental sample, as well as introduced during the MDA or sequencing process (Woyke *et al.*, 2011; Engel *et al.*, 2014; Field *et al.*, 2014). Similarly, metagenome binning and assembly often carries various errors and artifacts depending on the methods used (Nielsen *et al.*, 2014). Even cultured isolate genomes have been shown to lack immunity to contamination with other species (Parks *et al.*, 2014; Mukherjee *et al.*, 2015). As sequencing of these genome product types rapidly increases, contaminant sequences are finding their way into public databases as reference sequences. It is therefore extremely important to define standardized and automated protocols for quality control and decontamination, which would go a long way towards establishing quality standards for all microbial genome product types.

Current procedures for decontamination and quality control of genome sequences in single cells and metagenome bins are heavily manual and can consume hours/megabase when performed by expert biologists. Supervised decontamination typically involves homology-based inspection of ribosomal RNA sequences and protein coding genes, as well as visual analysis of k-mer frequency plots and guanine–cytosine content (Clingenpeel, 2015). Manual decontamination is also possible through the software SmashCell (Harrington *et al.*, 2010), which contains a tool for visual identification of contaminants from a self-organizing map and corresponding U-matrix. Another existing software tool, DeconSeq (Schmieder and Edwards, 2011), automatically removes contaminant sequences, however, the contaminant databases are required input. The former lacks automation, whereas the latter requires prior knowledge of contaminants, rendering both applications impractical for high-throughput decontamination.

Here, we introduce ProDeGe, the first fully automated computational protocol for decontamination of genomes. ProDeGe uses a combination of homology-based and sequence composition-based approaches to separate contaminant sequences from the target genome draft. It has been pre-calibrated to discard at least 84% of the contaminant sequence, which results in retention of a median 84% of the target sequence. The standalone software is freely available at http://prodege.jgi-psf.org//downloads/src and can be run on any system that has Perl, R (R Core Team, 2014), Prodigal (Hyatt *et al.*, 2010) and NCBI Blast (Camacho *et al.*, 2009) installed. A graphical viewer allowing further exploration of data sets and exporting of contigs accompanies the web application for ProDeGe at http://prodege.jgi-psf.org, which is open to the wider scientific community as a decontamination service (Supplementary Figure S1).

The assembly and corresponding NCBI taxonomy of the data set to be decontaminated are required inputs to ProDeGe (Figure 1a). Contigs are annotated with genes following which, eukaryotic contamination is removed based on homology of genes at the nucleotide level using the eukaryotic subset of NCBI's Nucleotide database as the reference. For detecting prokaryotic contamination, a curated database of reference contigs from the set of high-quality genomes within the Integrated Microbial Genomes (IMG; Markowitz *et al.*, 2014) system is used as the reference. This ensures that errors in public reference databases due to poor quality of sequencing, assembly and annotation do not negatively impact the decontamination process. Contigs determined as belonging to the target organism based on nucleotide level homology to sequences in the above database are defined as ‘Clean', whereas those aligned to other organisms are defined as ‘Contaminant'. Contigs whose origin cannot be determined based on alignment are classified as ‘Undecided'. Classified clean and contaminated contigs are used to calibrate the separation in the subsequent 5-mer based binning module, which classifies undecided contigs as ‘Clean' or ‘Contaminant' using principal components analysis (PCA) of 5-mer frequencies. This parameter can also be specified by the user. When data sets do not have taxonomy deeper than phylum level, or a single confident taxonomic bin cannot be detected using sequence alignment, solely 9-mer based binning is used due to more accurate overall classification. In the absence of a user-defined cutoff, a pre-calibrated cutoff for 80% or more specificity separates the clean contigs from contaminated sequences in the resulting PCA of the 9-mer frequency matrix. Details on ProDeGe's custom database, evaluation of the performance of the system and exploration of the parameter space to calibrate ProDeGe for a high accurate classification rate are provided in the Supplementary Material.

The performance of ProDeGe was evaluated using 182 manually screened SAGs (Figure 1b,Supplementary Table S1) from two studies whose data sets are publicly available within the IMG system: genomes of 107 SAGs from an Arabidopsis endophyte sequencing project and 75 SAGs from the Microbial Dark Matter (MDM) project* (only 75/201 SAGs from the MDM project had 1:1 mapping between contigs in the unscreened and the manually screened versions, hence these were used; Rinke *et al.*, 2013). Manual curation of these SAGs demonstrated that the use of ProDeGe prevented 5311 potentially contaminated contigs in these data sets from entering public databases. Figure 2a demonstrates the sensitivity vs specificity plot of ProDeGe results for the above data sets. Most of the data points in Figure 2a cluster in the top right of the box reflecting a median retention of 89% of the clean sequence (sensitivity) and a median rejection of 100% of the sequence of contaminant origin (specificity). In addition, on average, 84% of the bases of a data set are accurately classified. ProDeGe performs best when the target organism has sequenced homologs at the class level or deeper in its high-quality prokaryotic nucleotide reference database. If the target organism's taxonomy is unknown or not deeper than domain level, or there are few contigs with taxonomic assignments, a target bin cannot be assessed and thus ProDeGe removes contaminant contigs using sequence composition only. The few samples in Figure 2a that demonstrate a higher rate of false positives (lower specificity) and/or reduced sensitivity typically occur when the data set contains few contaminant contigs or ProDeGe incorrectly assumes that the largest bin is the target bin. Some data sets contain a higher proportion of contamination than target sequence and ProDeGe's performance can suffer under this condition. However, under all other conditions, ProDeGe demonstrates high speed, specificity and sensitivity (Figure 2). In addition, ProDeGe demonstrates better performance in overall classification when nucleotides are considered than when contigs are considered, illustrating that longer contigs are more accurately classified (Supplementary Table S1).

All SAGs used in the evaluation of ProDeGe were assembled using SPAdes (Bankevich *et al.*, 2012). In-house testing has shown that reads assembled with SPAdes from different strains or even slightly divergent species of the same genera may be combined into the same contig (Personal communications, KT and Robert Bowers). Ideally, the DNA in a well that gets sequenced belongs to a single cell. In the best case, contaminant sequences need to be at least from a different species to be recognized as such by the homology-based screening stage. In the absence of closely related sequenced organisms, contaminant sequences need to be at least from a different genus to be recognized as such by the composition-based screening stage (Supplementary Material). Thus, there is little risk of ProDeGe separating sequences from clonal populations or strains. We have found species- and genus-level contamination in MDA samples to be rare.

To evaluate the quality of publicly available uncultured genomes, ProDeGe was used to screen 185 SAGs and 14 GMGs (Figure 1b). Compared with CheckM (Parks *et al.*, 2014), a tool which calculates an estimate of genome sequence contamination using marker genes, ProDeGe generally marks a higher proportion of sequence as ‘Contaminant' (Supplementary Table S2). This is because ProDeGe has been calibrated to perform at high specificity levels. The command line version of ProDeGe allows users to conduct their own calibration and specify a user-defined distance cutoff. Further, CheckM only outputs the proportion of contamination, but ProDeGe actually labels each contig as ‘Clean' or ‘Contaminant' during the process of automated removal.

The web application for ProDeGe allows users to export clean and contaminant contigs, examine contig gene calls with their corresponding taxonomies, and discover contig clusters in the first three components of their k-dimensional space. Non-linear approaches for dimensionality reduction of k-mer vectors are gaining popularity (van der Maaten and Hinton, 2008), but we observed no systematic advantage of using t-Distributed Stochastic Neighbor Embedding over PCA (Supplementary Figure S2).

ProDeGe is the first step towards establishing a standard for quality control of genomes from both cultured and uncultured microorganisms. It is valuable for preventing the dissemination of contaminated sequence data into public databases, avoiding resulting misleading analyses. The fully automated nature of the pipeline relieves scientists of hours of manual screening, producing reliably clean data sets and enabling the high-throughput screening of data sets for the first time. ProDeGe, therefore, represents a critical component in our toolkit during an era of next-generation DNA sequencing and cultivation-independent microbial genomics.

## Figures and Tables

**Figure 1:**
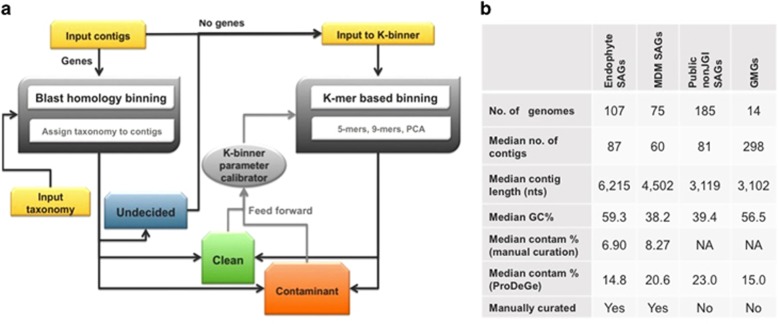
(**a**) Schematic overview of the ProDeGe engine. (**b**) Features of data sets used to validate ProDeGe: SAGs from the Arabidopsis endophyte sequencing project, MDM project, public data sets found in IMG but not sequenced at the JGI, as well as genomes from metagenomes. All the data and results can be found in Supplementary Table S3.

**Figure 2:**
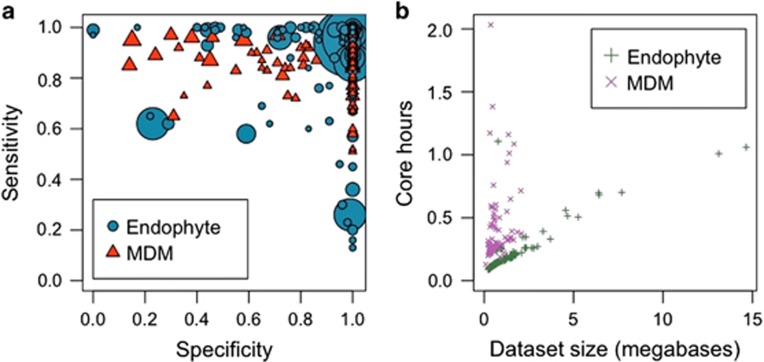
ProDeGe accuracy and performance scatterplots of 182 manually curated single amplified genomes (SAGs), where each symbol represents one SAG data set. (**a**) Accuracy shown by sensitivity (proportion of bases confirmed ‘Clean') vs specificity (proportion of bases confirmed ‘Contaminant') from the Endophyte and Microbial Dark Matter (MDM) data sets. Symbol size reflects input data set size in megabases. Most points cluster in the top right of the plot, showing ProDeGe's high accuracy. Median and average overall results are shown in Supplementary Table S1. (**b**) ProDeGe completion time in central processing unit (CPU) core hours for the 182 SAGs. ProDeGe operates successfully at an average rate of 0.30 CPU core hours per megabase of sequence. Principal components analysis (PCA) of a 9-mer frequency matrix costs more computationally than PCA of a 5-mer frequency matrix used with blast-binning. The lack of known taxonomy for the MDM data sets prevents blast-binning, thus showing longer finishing times than the endophyte data sets, which have known taxonomy for use in blast-binning.
