# Supplementary material for: ProDeGe: a computational protocol for fully automated decontamination of genomes
Source: ISME J. 2015 Jun 9;10(1):269–72. doi: 10.1038/ismej.2015.100 (PMC4681846; doi:10.1038/ismej.2015.100)
Supplement: Supplementary Material [file ismej2015100x1.doc]

**SUPPLEMENTARY MATERIAL**

**Supplementary Materials and Methods**

**ProDeGe Nucleotide Databases**

ProDeGe utilizes two custom databases in the blast-binning step. ProDeGe’s IMG prokaryotic database is 90G in size and consists of 25,073 high-quality public genomes with 2,247,550 nucleotide sequences. The database includes 2 domains (bacteria and archaea), 59 phyla, 71 classes, 148 orders, 328 families, 1,474 genera, and 6,651 species. The utilization of high quality curated reference sequences from IMG for the detection of bacterial/archaeal contamination minimizes the propagation of database errors. ProDeGe’s eukaryotic database is 28G in size and consists of 10,422,852 sequences from NCBI’s Nucleotide database.

**ProDeGe algorithm formation and performance evaluation**

The criteria used for parameter determination was the proportion of bases that ProDeGe accurately classifies, while also looking at sensitivity and specificity. For Blast homology, we use the default e-value cutoff of 10 and filter the results with the criteria that the hit must cover 50% or more of the query gene with over 30 percent identity. This ensures the retention of only high-quality hits. The taxonomy the user inputs can greatly affect the results of ProDeGe. The deepest taxonomy known should always be used as input, as it results in the most accurate decontamination. We show extensive analysis of different k-mers in Supplementary Tables S4 and S5. k=5 was chosen for datasets in which blast-binning was successful, resulting in a median overall classification success of 95%. k=9 was chosen for datasets in which blast-binning was not successful, resulting in a median overall classification success of 80%. When contigs cannot be identified from the target organism, thus setting a k-mer anchor for the classification, automatic decontamination becomes a much more difficult problem to solve and thus results in less successful classification.

To study the effect of ProDeGe’s k-mer binning performance on genus-level contamination, we chose a manually curated dataset of the genus Burkholderia from the Arabidopsis endophyte project that had significant contamination with the genus Ralstonia. Burkholderia sp. JGI 001021-E08 (IMG Taxon ID 2528768045) contained 545 sequences before manual curation, which resulted in only 62 sequences identified from the target cell. Using both blast- and kmer-binning, ProDeGe correctly classified 89% of the base pairs from the target and 99% of the base pairs of contaminant origin. We turned blast-binning off and ran ProDeGe again using only kmer-binning. ProDeGe correctly classified 21% of the base pairs from the target and 70% of the base pairs of contaminant origin. 59 contigs from the contaminant Ralstonia were classified as clean. ProDeGe kmer-binning without the homology based step does not easily separate sequences at the genus level, and thus is not able to classify at a lower level. Blast-binning does not classify contigs past the species level. Thus, there is little risk of ProDeGe separating sequences from clonal populations or strains.

**Supplementary Tables and Figures**

**
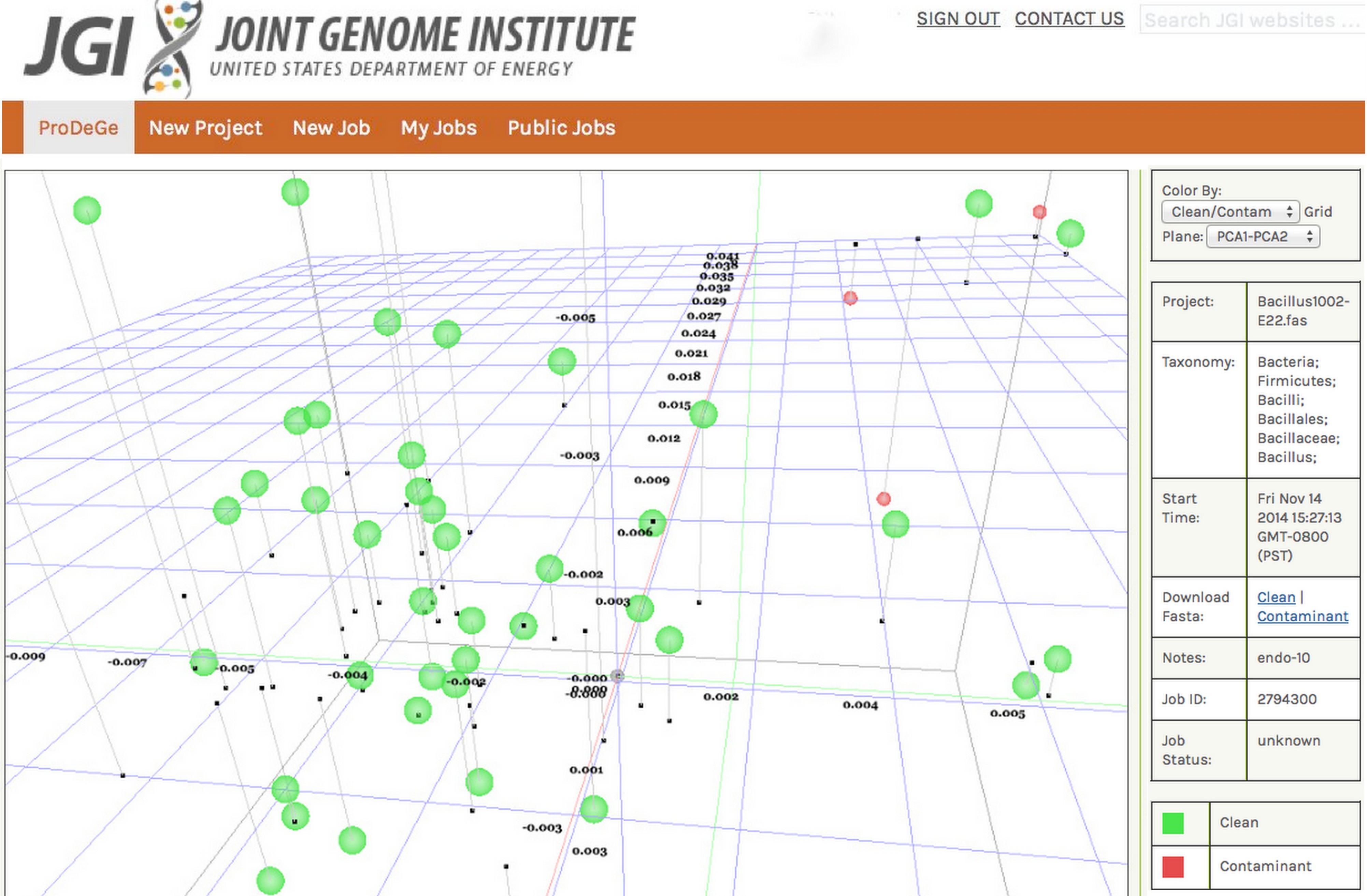
**

**Figure S1** Visualization of the SAG dataset Bacillus sp. JGI 0001002-E22 (IMG Taxon OID 2528768030) shown using ProDeGe’s web-based version. Visit http://prodege.jgi-psf.org.


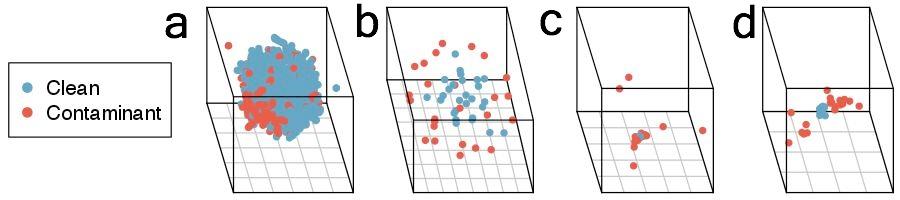


**Figure S2** 3-D plots of dataset Marinimicrobia bacterium JGI 0000077-B04 (IMG Taxon OID 2264867239) **(a)** Dimensionality reduction using t-SNE, with one point representing the 4-mer profile of 5,000 base pairs of a contig (step length 500). **(b)** Using t-SNE, with one point representing one contig’s 7-mer profile **(c)** Dimensionality reduction using Principal Components Analysis, with one point representing one contig’s 9-mer profile. **(d)** A magnified view of plot (c) which shows the blue points in a very tight cluster. We found that PCA performed better at separating the clean from contaminant contigs, which is shown in this example as the red and blue dots are intermixed in plots (a) and (b) but clearly separate in plots (c) and (d).

**Table S1** Proportion of clean sequence retained (sensitivity), proportion of contaminant sequence discarded (specificity), and overall correct classification by ProDeGe for the MDM and Endophyte datasets plotted in Figure 2.

| **Dataset (sample size)** | | **MDM (75)** | **Endophyte (107)** |
| --- | --- | --- | --- |
| Median Sensitivity | Counting bases | 0.86 | 0.95 |
|  | Counting contigs | 0.53 | 0.88 |
| Mean Sensitivity (standard deviation) | Counting bases | 0.83 (0.11) | 0.85 (0.21) |
|  | Counting contigs | 0.53 (0.14) | 0.77 (0.24) |
| Median Specificity | Counting bases | 1.00 | 1.00 |
|  | Counting contigs | 1.00 | 1.00 |
| Mean Specificity (standard deviation) | Counting bases | 0.81 (0.25) | 0.85 (0.24) |
|  | Counting contigs | 0.95 (0.08) | 0.88 (0.20) |
| Median Overall Correct Classification | Counting bases | 0.83 | 0.95 |
|  | Counting contigs | 0.70 | 0.88 |
| Mean Overall Correct Classification (standard deviation) | Counting bases | 0.80 (0.14) | 0.86 (0.18) |
|  | Counting contigs | 0.69 (0.10) | 0.81 (0.18) |

**Table S2** ProDeGe genome sequence contamination percentage compared to CheckM. Unlike ProDeGe, CheckM results do not specify which contig is from contaminant origin. Contigs mis-classified by ProDeGe as “Contaminants” are very short in length and constitute a tiny fraction of the total bases in a dataset.

| **IMG Taxon OID** | **Genome Name** | **Genome Size (bp)** | **Number of contigs** | **ProDeGe contam-**  **ination %** | **CheckM contam-ination %** |
| --- | --- | --- | --- | --- | --- |
| 2264867108 | Cloacimonetes bacterium SCGC AAA252-G07 (SAK_001_50) | 499,464 | 60 | 34.70 | 4.17 |
| 2264867132 | Hydrogenedentes bacterium JGI 0000039-J10 (TAsludge_001_156) | 356,599 | 30 | 14.97 | 4.17 |
| 2264867141 | Parcubacteria bacterium SCGC AAA011-N16 (Dusel_001_262) | 431,116 | 43 | 27.21 | 4.17 |
| 2264867158 | Omnitrophica bacterium SCGC AAA257-O07 (Etoliko_001_147) | 767,845 | 60 | 23.55 | 5.32 |
| 2264867177 | Aminicenantes bacterium SCGC AAA252-D18 (SAK_001_40) | 1,408,933 | 99 | 17.25 | 4.58 |
| 2264867202 | Atribacteria bacterium SCGC AAA255-G05 (SAK_001_130) | 1,653,325 | 131 | 24.82 | 3.99 |
| 2527291509 | Aigarchaeota archaeon SCGC AAA471-B22 (Combined_Assembly_pSL4_1__pSL4) | 1,180,338 | 128 | 36.87 | 5.09 |
| 2527291514 | Calescamantes bacterium JGI 0000106-G12 (Combined_Assembly_EM19_1__EM19) | 2,317,901 | 146 | 16.86 | 6.87 |
| 2527291517 | Cloacimonetes bacterium SCGC AAA252-E13 (Combined_Assembly_KSB1_1__KSB1) | 3,070,162 | 193 | 16.83 | 21.54 |
| 2527291526 | Marinimicrobia bacterium JGI 0000039-D08 (Combined_Assembly_SAR406_1__SAR406) | 2,357,096 | 112 | 8.59 | 11.29 |

**Table S3**

Separate document Supplementary Table S3.

**Table S4**: Exploration of parameter space by adjusting k-mer size for the Endophyte datasets, using calibrated cutoff. Sensitivity is defined as the proportion of bases that are truly from the target organism that ProDeGe classified as such. Specificity is defined as the proportion of bases that are of contaminant origin that ProDeGe classified as such. k=5 gives a slight advantage for accurately classifying the datasets.

| Sensitivity | Min | 1st Qu | Median | Mean | 3rd Qu | Max |
| --- | --- | --- | --- | --- | --- | --- |
| k=4 | 0.12 | 0.76 | 0.93 | 0.84 | 0.98 | 1.00 |
| **k=5** | **0.13** | **0.79** | **0.95** | **0.85** | **0.99** | **1.00** |
| k=6 | 0.13 | 0.79 | 0.95 | 0.85 | 0.99 | 1.00 |
| k=7 | 0.13 | 0.78 | 0.94 | 0.85 | 0.99 | 1.00 |

| Specificity | Min | 1st Qu | Median | Mean | 3rd Qu | Max |
| --- | --- | --- | --- | --- | --- | --- |
| **k=4** | **0.00** | **0.82** | **1.00** | **0.86** | **1.00** | **1.00** |
| k=5 | 0.00 | 0.79 | 1.00 | 0.85 | 1.00 | 1.00 |
| k=6 | 0.00 | 0.77 | 1.00 | 0.85 | 1.00 | 1.00 |
| k=7 | 0.00 | 0.71 | 1.99 | 0.83 | 1.00 | 1.00 |

| Proportion of bases correctly classified | Min | 1st Qu | Median | Mean | 3rd Qu | Max |
| --- | --- | --- | --- | --- | --- | --- |
| k=4 | 0.17 | 0.82 | 0.95 | 0.85 | 0.98 | 1.00 |
| **k=5** | **0.17** | **0.83** | **0.95** | **0.86** | **0.98** | **1.00** |
| k=6 | 0.17 | 0.82 | 0.95 | 0.86 | 0.97 | 1.00 |
| k=7 | 0.17 | 0.82 | 0.94 | 0.86 | 0.97 | 1.00 |

**Table S5**: Exploration of parameter space by adjusting k-mer size for the MDM datasets, using best default cutoff. k=9 gives a slight advantage for accurately classifying the datasets. At k=12, R ran out of memory in the process of performing PCA and had unacceptably long runtime. Above k=9, there was not an appreciable improvement in performance.

| Sensitivity | Min | 1st Qu | Median | Mean | 3rd Qu | Max |
| --- | --- | --- | --- | --- | --- | --- |
| k=6 | 0.00 | 0.59 | 0.74 | 0.69 | 0.86 | 0.98 |
| k=7 | 0.13 | 0.73 | 0.85 | 0.80 | 0.90 | 0.98 |
| k=8 | 0.23 | 0.76 | 0.86 | 0.83 | 0.91 | 0.98 |
| **k=9** | **0.51** | **0.77** | **0.86** | **0.83** | **0.91** | **0.98** |
| k=10 | 0.42 | 0.74 | 0.85 | 0.82 | 0.90 | 0.98 |

| Specificity | Min | 1st Qu | Median | Mean | 3rd Qu | Max |
| --- | --- | --- | --- | --- | --- | --- |
| **k=6** | **0.14** | **1.00** | **1.00** | **0.95** | **1.00** | **1.00** |
| k=7 | 0.14 | 1.00 | 1.00 | 0.91 | 1.00 | 1.00 |
| k=8 | 0.14 | 0.71 | 1.00 | 0.85 | 1.00 | 1.00 |
| k=9 | 0.14 | 0.67 | 1.00 | 0.82 | 1.00 | 1.00 |
| k=10 | 0.14 | 0.67 | 1.00 | 0.82 | 1.00 | 1.00 |

| Proportion of bases correctly classified | Min | 1st Qu | Median | Mean | 3rd Qu | Max |
| --- | --- | --- | --- | --- | --- | --- |
| k=6 | 0.13 | 0.64 | 0.76 | 0.73 | 0.86 | 0.98 |
| k=7 | 0.21 | 0.73 | 0.82 | 0.80 | 0.87 | 0.98 |
| k=8 | 0.21 | 0.74 | 0.83 | 0.80 | 0.88 | 0.98 |
| **k=9** | **0.21** | **0.76** | **0.83** | **0.80** | **0.89** | **0.98** |
| k=10 | 0.21 | 0.73 | 0.83 | 0.79 | 0.88 | 0.98 |
